# Recent advances in Merkel cell carcinoma

**DOI:** 10.12688/f1000research.20747.1

**Published:** 2019-11-26

**Authors:** Caitlin G. Robinson, Daniel Tan, Siegrid S. Yu

**Affiliations:** 1 Colorado Springs Dermatology Clinic, 170 South Parkside Drive, Colorado Springs, CO, 80910, USA; 2 Department of Dermatology, University of California San Francisco, 1701 Divisadero Street, San Francisco, CA, 94115, USA

**Keywords:** Merkel cell carcinoma, abscopal effect, immunotherapy

## Abstract

Merkel cell carcinoma (MCC) is a rare and aggressive neuroendocrine skin cancer that has been historically associated with limited treatment options and poor prognosis. In the past 10 years, research in MCC has progressed significantly, demonstrating improved outcomes when treating with immunotherapy, particularly PD-1/PD-L1 inhibitors, when compared with conventional chemotherapy. There is also increasing evidence of the abscopal effect, a phenomenon describing the regression of untreated, distant MCC tumors following local radiation therapy. Additionally, antibodies to Merkel cell polyomavirus oncoproteins have been found to correlate with disease burden in a subset of patients, providing a useful tool for surveillance after treatment. Guidelines for the management of MCC will likely continue to change as research on surveillance and treatment of MCC continues.

## Introduction

Merkel cell carcinoma (MCC) is a rare and aggressive neuroendocrine skin cancer occurring most commonly in elderly white men and typically arising in sun-exposed areas of the head and neck ^1^. The mean age of patients at diagnosis is 70 years, and risk factors include immunosuppression and ultraviolet radiation exposure ^2^. More than 2500 cases of MCC are diagnosed each year in the United States, and the incidence has increased steadily since the early 1990s by 5% to 10% per year, resulting in an approximately 5.4-fold increase over the course of 18 years ^3, 4^. These tumors are associated with a high mortality rate due to rapid growth and metastasis, as 6% to 16% of cases are already stage IV at the time of diagnosis ^3^.

The staging of MCC relies on the physical exam, sentinel lymph node biopsy, and imaging studies. Treatment regimen is dependent on staging and typically consists of combinations of surgery, radiation, or immunotherapy. Completion lymph node dissection or radiation therapy is indicated for patients with a positive sentinel lymph node biopsy ^3^. Historically, conventional chemotherapy was administered to patients with advanced disease, and although 53% to 76% of MCCs are initially responsive to chemotherapy, responses lacked durability: progression-free survival was limited (1.9 to 4.6 months) ^5, 6^. This modality of treatment is now limited to palliative treatment for patients who are not candidates for immunotherapy. Prior five-year overall survival rates were 55% for local disease, 35.4% for nodal disease, and 13.5% for distantly metastatic disease ^7^.

Recent advances in treatment and surveillance are now providing hope for patients with advanced disease. Similar to recent developments in the treatment of melanoma, immunotherapy has improved patient outcomes by providing effective and durable responses for some patients with MCC. Local radiation therapy has been shown to augment this response to immunotherapies, even at sites distant from the field of radiation, a phenomenon known as the abscopal effect. Also, monitoring of antibodies to the Merkel cell polyomavirus (MCPyV) is making it easier for physicians to anticipate and monitor for recurrence. We will explore these latest advances herein.

## Use of antibodies to Merkel cell polyomavirus antigens

In 2008, Feng *et al*. first identified the MCPyV integrated into the DNA of 8 of 10 MCC tumors ^8^, making it the first polyomavirus to be considered a causal agent of a human cancer. Although 60% of the general population possesses antibodies indicating prior infection ^9^, MCC remains a very rare cancer, indicating that virus-induced tumorigenesis occurs infrequently. The discovery of this polyomavirus was the first step toward the recent development of viral antibodies used as biomarkers in the virus-positive subset of patients with MCC.

The major families of genes in MCPyV are the tumor-associated antigens and the capsid genes. The tumor-associated antigens—the small T (ST) and large T (LT) antigens—act as the major oncoproteins of the virus. The mechanism of tumorigenesis of these oncoproteins is not fully understood, although evidence does suggest that expression of both T antigens is necessary for survival and replication of virus-positive MCC cells ^10– 13^. Antibodies to the major capsid protein (VP1) are found in nearly all patients with MCC as well as in 42% to 77% of the general population, whereas antibodies to the T antigens are specific to patients with MCC ^9^. Virus-positive MCC is identified by the detection of antibodies to these MCPyV T antigens, whereas virus-negative patients lack these antibodies. Virus-positive MCC accounts for about 80% to 90% of MCCs in North America; however, this rate varies worldwide ^9, 14^.

Recent studies have investigated the utility of MCPyV antibodies in predicting prognosis and surveillance for recurrence. In 2010, a study published by Paulson *et al*. first demonstrated that an increase in antibodies to MCPyV T antigens could predict and sometimes precede clinical detection of recurrent disease in patients with virus-positive disease ^15^. A 2016 study by Samimi *et al*. aimed to determine whether baseline and follow-up antibody titers were reflective of prognosis in patients with MCC ^12^. It was found that low VP1 antibodies at baseline corresponded to higher risk of recurrence and increased mortality but did not vary with disease burden. Titers of antibodies to T antigens at baseline did not indicate prognosis; however, an increase in these antibodies did correlate with recurrence ^12^. In 2017, Paulson *et al*. published results of a prospective validation study including 219 patients with newly diagnosed MCC, demonstrating that a rising T antigen antibody holds a positive predictive value for increasing tumor burden of 66% to 83% but that a falling antibody level has a negative predictive value of 97%. MCPyV-antibody positivity at baseline corresponded to a 42% decrease in risk of recurrence ^9^.

Given the evidence in support of the MCPyV antibodies as biomarkers and prognostic indicators, it may be useful to draw baseline antibody levels on patients at the time of MCC diagnosis to help guide surveillance. A low baseline VP1 antibody titer may indicate a need for heightened surveillance with regular imaging, and serial T antigen antibody titers can be useful in monitoring for recurrence. For patients with high baseline antibodies, drawing serial titers for surveillance has the added benefit of sparing radiation exposure associated with routine follow-up imaging. The lab at the University of Washington has a Merkel cell assay that is currently available and that includes a baseline VP1 test and tests for antibodies to the ST antigen. ST antigen antibodies appear to be slightly more specific for MCC, as antibodies to LT antigens are found, albeit rarely, in general population controls, indicating a possible cross-reaction with another protein ^15^.

## Immunotherapy in Merkel cell carcinoma

Over the past decade, research has shown that blockade of the PD-1/PD-L1 pathway is associated with improved overall survival and durability of response in cases of advanced MCC ^16^. Since 2017, US Food and Drug Administration (FDA) approval has been granted for both the PD-L1 inhibitor avelumab and the PD-1 inhibitor pembrolizumab in the treatment of metastatic MCC in patients age 12 or older. Though not yet FDA-approved to treat MCC, the PD-1 inhibitor nivolumab is also recommended for the treatment of advanced MCC under current National Comprehensive Cancer Network guidelines. Cytotoxic chemotherapy regimens are not recommended unless a contraindication to immunotherapy is present ^3^.

The PD-L1 inhibitor avelumab is a fully human IgG1 antibody that targets PD-L1, preventing its interaction with the PD-1 receptor ^17^. FDA approval for the treatment of MCC was granted on the basis of the JAVELIN Merkel 200 trial, a phase 2 prospective, open-label, multicenter trial including patients with metastatic MCC that had failed conventional chemotherapy. The most recently published data at 2 years reflected a 33% objective response rate. Durable responses were noted with stable progression-free survival at 26% to 29% over the first 2 years. When the trial was later opened to treatment-naïve patients, objective response rate increased to 62.1% (13.8% complete response and 48.3% partial response) ^18^.

The PD-1 inhibitors nivolumab and pembrolizumab are also being extensively studied for treatment of MCC, and thus far results have been similar to those of avelumab with regard to response rate, durability, and safety profile. Objective response rates range from 53% to 56% for pembrolizumab ^19, 20^ to 45% to 65% for nivolimumab ^7, 21^. Durable responses have been noted with both ^19, 21^. In a study by Nghiem *et al*. of patients with metastatic/recurrent MCC treated with pembrolizumab, 24-month progression-free survival was 48%, demonstrating a durable response to treatment ^19^. In general, all discussed PD-1/PD-L1 inhibitors appear to be well tolerated with minimal side effects, and rates of grade 3 or 4 adverse events occurred in 5% to 21% ^3, 7, 18, 21^.

A frequently investigated aspect of recent MCC research has focused on treatment response rates of virus-negative versus virus-positive MCCs. In general, most skin cancers have high tumor mutational burdens (TMBs) whereas virus-positive skin cancers, such as most MCCs, tend to have very low mutational burdens. Although TMBs often correlate with greater response rates to immunotherapies, virus-positive MCC is often responsive to anti–PD-1 immunotherapies and this may be due to antigenicity provided by expression of viral proteins. It is thought that both skin cancers with high TMBs and virus-associated skin cancers have the potential to be responsive to immunotherapies because of their expression of viable targets: neoantigens and viral proteins, respectively ^4^. Furthermore, although virus-negative MCC is associated with worsened prognosis and differs in its tumor-specific immunity ^9, 12, 22^, both virus-positive and virus-negative MCCs have shown favorable responses to immune checkpoint therapies regardless of PD-L1 expression ^17, 19^.

Many ongoing clinical trials are focusing on combination or experimental treatments for MCC. These treatments include ipilimumab, tremelimumab/durvalumab, adoptive T-cell transfer, natural killer cell infusions, MLN0128, tyrosine kinase inhibitors (cabozantanib), peptide receptor radionuclide therapy (PPRT), and talimogene laherparepvec (T-VEC). Another trial is investigating tailored treatments based on each patient’s specific tumor genomics ^16, 17^.

Although most MCC studies focus on immunocompetent patients, immunosuppression confers a more dismal prognosis and limits treatment options. A case series by Tarabadkar *et al*. included one patient with metastatic MCC on systemic immunosuppressive therapy for psoriatic arthritis ^23^. For 5 months after initiating treatment with the tyrosine kinase inhibitor pazopanib, this patient showed clinical improvement in MCC tumor burden, and immunosuppressants were discontinued because of improvement in arthritis symptoms ^23^. Of the immunosuppressed patients, organ transplant recipients have the most limited treatment options because of risk of transplant rejection on immunotherapy. One clinical trial is currently investigating combination tacrolimus/prednisone and nivolumab/ipilimumab in kidney transplant patients with metastatic solid tumors (ClinicalTrials.gov Identifier: NCT03816332). Further studies are needed to identify safe alternative treatments, such as targeted therapies, for this subset of patients.

## The abscopal effect in the treatment of Merkel cell carcinoma

One of the more interesting developments in the recent MCC literature has been the realization of the abscopal effect, a phenomenon describing the regression of distant, untreated metastatic tumors following local radiation therapy. The mechanism is hypothesized to be increased antigen exposure and presentation and subsequent CD8 ^+^ recruitment following radiation, improved functioning of T cells, and direct tumor debulking by removing the inhibition on CD8 ^+^ T cells ^24– 26^. Further literature points to an increased rate of the abscopal effect when radiation is used concurrently with immune checkpoint inhibitors, suggesting synergistic effects ^25^. The abscopal effect has also been noted in the treatment of other solid tumors ^27^.

A case presented by Cotter *et al*. in 2011 demonstrated the abscopal effect in a patient with several cutaneous MCC metastases on the right lower extremity ^24^. All but two lesions were treated with radiation, yet all of the lesions, including two untreated lesions on the right ankle outside of the field of radiation, responded. The patient remained clear of metastatic disease for 25 months ^24^. A case series by Xu *et al*. in 2018 reported two cases with advanced MCC progressing on pembrolizumab for which palliative localized radiation was given in a single fraction, resulting in a decrease in tumor burden appreciable at 2 to 4 months after radiation and a continued decrease in overall tumor burden over the course of 12 months ^25^. Complete remission was achieved for at least 17 months ^25^. Yet another case report, by Bloom *et al*. in 2019, described the use of adjuvant, localized radiation to the groin of a 70-year-old man with recurrent and progressive metastatic MCC in conjunction with pembrolizumab therapy, resulting in complete response at 3 months after treatment, including clearance of metastases outside of the field of radiation ^26^. This response was durable for at least 20 months after radiation ^26^.

Research investigating the benefit of combining radiation treatment with immunotherapy is ongoing. Nonetheless, employing the abscopal effect could be a viable treatment option for patients with unresectable advanced MCC that is not adequately responding to current treatments. Several clinical trials are being conducted for MCC by using radiation in conjunction with PD-1 inhibitors as well as avelumab and the CTLA-4 inhibitor ipilimumab.

## Conclusions

Although there is a long road ahead to achieving successful treatment of MCCs, the past decade has marked a turning point in the understanding, care, and prognosis of this rare skin cancer. According to ClinicalTrials.gov, 46 clinical trials on MCC were ongoing or enrolling at the time of writing and many of them focus on novel or combination treatments. We will likely witness changes to the treatment guidelines as this literature continues to grow. Nonetheless, when compared with the dismal outlook that accompanied a diagnosis of MCC 10 years ago, the future is looking brighter for patients with MCC.
